# 450 million years in the making: mapping the evolutionary foundations of germinal centers

**DOI:** 10.3389/fimmu.2023.1245704

**Published:** 2023-08-11

**Authors:** Hanover Matz, Helen Dooley

**Affiliations:** Department of Microbiology and Immunology, Institute of Marine and Environmental Technology, University of Maryland School of Medicine, Baltimore, MD, United States

**Keywords:** germinal center, antibody, affinity maturation, secondary lymphoid organs, B cells, T follicular helper cells, follicular dendritic cells, evolution

## Abstract

Germinal centers (GCs) are distinct microanatomical structures that form in the secondary lymphoid organs of endothermic vertebrates (i.e., mammals and some birds). Within GCs, B cells undergo a Darwinian selection process to identify clones which can respond to pathogen insult as well as affinity mature the B cell repertoire. The GC response ultimately generates memory B cells and bone marrow plasma cells which facilitate humoral immunological memory, the basis for successful vaccination programs. GCs have not been observed in the secondary lymphoid organs of ectothermic jawed vertebrates (i.e., fishes, reptiles, and amphibians). However, abundant research over the past decades has indicated these organisms can produce antigen specific B cell responses and some degree of affinity maturation. This review examines data demonstrating that the fundamentals of B cell selection may be more conserved across vertebrate phylogeny than previously anticipated. Further, research in both conventional mammalian model systems and comparative models raises the question of what evolutionary benefit GCs provide endotherms if they are seemingly unnecessary for generating the basic functional components of jawed vertebrate humoral adaptive immune responses.

## Introduction

B cells are the functional centerpiece of humoral adaptive immunity in jawed vertebrates. During the immune response, the host immune system selects B cell clones with B cell receptors (BCRs)/immunoglobulins (Igs) that recognize immunogens or antigens. These clones will proliferate and differentiate into antibody-secreting plasma cells, resulting in protective antibody titers that circulate in the blood and penetrate tissues to clear offending pathogens. In mammals and birds this response is facilitated by specialized microanatomical structures known as germinal centers (GCs). Over the course of a humoral response, GCs mediate maturation of peripheral antibody binding affinity (1, 2) and export effector cell types that generate long term immunological memory, namely long-lived bone marrow plasma cells (BMPCs) and memory B cells (MBCs) (3, 4).

GCs form in the secondary lymphoid organs (SLOs) after cognate B cells and T cells recognize antigen and interact at the follicular border (5, 6). Several functional characteristics define the GC reaction. B cells segregate into two poles or zones of the GC: the dark zone (DZ) containing cells known as centroblasts, and the light zone (LZ) containing cells known as centrocytes (7, 8). This compartmentalization is established by expression of specific chemokines and chemokine receptors. GC B cells expressing the receptor CXCR4 migrate to the DZ by sensing CXCL12 produced by stromal cells, while B cells expressing CXCR5 migrate towards CXCL13 produced in the LZ (7). Within the DZ B cells proliferate and express activation induced cytidine deaminase (AICDA) which mediates somatic hypermutation (SHM) of Ig variable (V) regions (2, 9). These clones can then exit the DZ and enter the LZ to undergo selection against antigen presented on the surface of follicular dendritic cells (FDCs) (7, 10). FDCs retain non-degraded antigen in immune complexes (ICs) (10) which preserve the non-linear epitopes BCRs can recognize. If SHM results in BCRs with increased binding affinity, the corresponding GC B cell clones can more efficiently extract antigen from the surface of FDCs (11, 12) and present antigenic peptides to T follicular helper (Tfh) cells present in the GC (13). Tfh cells supply signals that promote the survival of higher affinity clones (14, 15). Thus, through iterative rounds of proliferation/SHM and Darwinian selection, the GC reaction both identifies B cells which can bind antigens and supports affinity maturation of the polyclonal antibody response.

Bona fide GCs have only been observed in the SLOs of endothermic jawed vertebrates (mammals and birds); no ectothermic vertebrates studied have histologically observable GCs (16). The absence of GC structures in ectothermic vertebrates would suggest these taxa are incapable of generating effective B cell responses. Indeed, affinity maturation of antibodies in ectothermic vertebrates appears limited (approximately 10-fold maximum increase) (17–20) compared to endotherms (>100-fold increases) (1). Despite this, many studies demonstrate that ectothermic vertebrate antibody titers increase in an antigen-specific fashion in response to immunization or pathogen insult (18, 20–23), indicative of B cell clonal selection. AICDA-mediated SHM of Igs is also found in all jawed vertebrate lineages (20, 24–27), demonstrating a key role for mutation of BCRs in adaptive immunity. Finally, there is evidence for secondary recall responses in ectothermic jawed vertebrates (21, 28–30), indicative of B cell memory (although recall titers do not always exceed those of the primary response). Indeed, in some taxa immunological memory has been shown to persist for significant periods (>8 years) after primary exposure (31). Together these results demonstrate that ectothermic jawed vertebrate B cell responses are functionally protective in the absence of true GCs, even if affinity maturation is not as robust.

Interestingly, many functional characteristics deemed integral to mammalian B cell responses have since been shown to develop independently of the GC reaction. B cell clones which have undergone class switch recombination (CSR) of Ig isotypes, once believed to occur primarily within the GC, have been identified prior to GC-entry (32). Studies in mice indicate that affinity maturation of antibodies can proceed without GCs (33), and that extrafollicular B cell responses can facilitate both selection and affinity maturation (34). Finally, some evidence suggests that GCs may not be necessary for generating long-lived BMPCs (35, 36), and certainly memory B cells can be produced in a GC-independent fashion (37). Thus, if GCs are unnecessary for affinity maturation and immunological memory, and if ectothermic vertebrates can seemingly produce protective humoral responses without GCs, why did endothermic vertebrates evolve these intricately organized structures of B cell selection?

This review will examine what is known about B cell selection sites in jawed vertebrate lineages outside of mammals. Comparisons across taxa show that many fundamental components of the mammalian GC response are more conserved across jawed vertebrate phylogeny than previously appreciated.

## Mammals: germinal centers are forever

**Figure 1:**
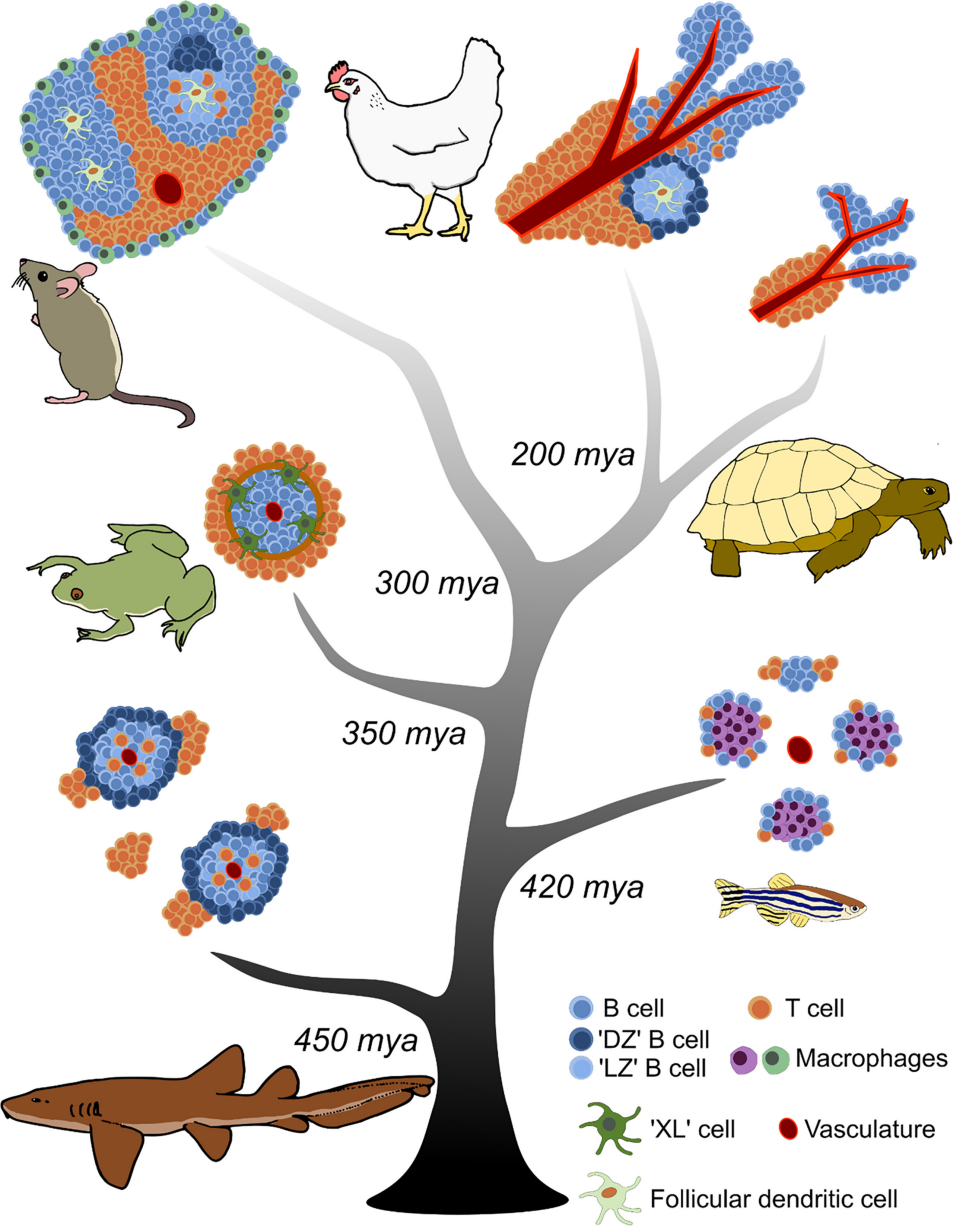
Evolution of splenic white pulp in jawed vertebrates. Increasing complexity in the organization of adaptive lymphocytes is observed from cartilaginous fishes to mammals and birds. The central tree represents the timeline of jawed vertebrate evolution; major branch points when taxonomic groups diverged from a common ancestor are indicated as millions of years ago (mya). Each jawed vertebrate lineage is represented adjacent to its branch by a depiction of the predominant comparative model species. Cell types and structures are annotated in the key.

Our understanding of the tissue architecture which supports B cell selection in mammals is derived primarily from studying the SLOs of mice (*Mus musculus*), specifically the C57BL/6 laboratory strain. The primordial SLO is the spleen, where antigen collects from the circulatory system and is brought into contact with the major cellular participants of the adaptive immune response: B cells, T cells, and antigen presenting cells (APCs). The spleen is present in all jawed vertebrates; in contrast, lymph nodes as SLOs for screening antigen in the afferent lymph are only observed in mammals and, reportedly, at least some avian species (38). Broadly, splenic immune architecture increases in complexity from basal jawed vertebrates, such as cartilaginous fishes, to those, like mammals, that emerged more recently (**Figure 1**) (reviewed in (39)). While the mouse spleen is not representative of every aspect of SLO architecture in mammals, or even exactly the same as the human spleen (reviewed in 40), it provides a good foundation for understanding mammalian B cell selection.

The spleen consists of red pulp and white pulp regions. Immune cells are primarily localized in the white pulp. The red pulp filters aged and dead red blood cells, and helps transport antigen to the white pulp (reviewed in (40)). The mouse splenic white pulp is demarcated from the red pulp by the marginal zone (**Figure 1**). The marginal zone contains multiple cell types that participate in antigen trafficking, including innate-like marginal zone B cells, marginal zone macrophages, and marginal metallophilic macrophages (41). The white pulp is permeable to small (<65 kDa) molecules, but larger antigens must be transported across the marginal zone to concentrate with cognate B and T cells (42). Embedded within the mouse white pulp are B cell follicles surrounded by a defined T cell zone, also known as the periarteriolar lymphoid sheath (PALS). Maintenance of the T cell zone is controlled by expression of the chemokine receptor CCR7 and corresponding ligands CCL19 and CCL21 produced by stromal cells (43, 44). B cells are localized to the B cell zone by expression of the chemokine receptor CXCR5 and its corresponding ligand CXCL13, produced by FDCs to maintain the structure of the follicle (45, 46). FDCs are specialized APCs derived from a nonhematopoietic precursor (47), are dependent on LTα1β2 signaling (48, 49), and express the cellular adhesion molecules VCAM-1 and ICAM-1, plus complement receptors CR1 and CR2 (50, reviewed in 51). Thus, the mammalian splenic architecture maintains clearly defined B and T cell zones separated from the surrounding red pulp in which B cell selection is orchestrated (**Figure 1**).

B cell selection occurs when B cells recognize their cognate antigens, migrate to the border of the B and T cell zones, receive T cell help, then proceed to form GCs in the B cell follicles (5, 6). It is within these specialized structures that four major processes occur: 1. Segregation of B cell clones into distinct functional zones based on chemokine receptor expression, 2. Presentation of nondegraded antigen in ICs by specialized APCs to B cells, 3. Proliferation of activated B cells and SHM of Ig V regions mediated by AICDA, and 4. Co-stimulation of B cells by Tfh cells. The combination of these processes results in Darwinian selection of antigen-specific B cells that subsequently affinity matures the peripheral antibody response. Mammalian B cells can express multiple Ig heavy (H) chain isotypes serving different effector functions: IgM, secreted as a high avidity pentamer; IgD, an enigmatic isotype expressed on the surface of naïve B cells; IgG, with various subclasses depending on the species; IgA, secreted as a dimer at mucosal barriers; and IgE, typically associated with defenses against helminth infections (reviewed in 52).

While placental mammals (Eutheria) arose ~160 million years ago (mya) (53), the ‘mammalian model’ of B cell selection is built upon ~500 million years of prior jawed vertebrate evolutionary history. Indeed, while many features of the mammalian GC reaction were long assumed to be exclusive characteristics, examination of other jawed vertebrate lineages reveals analogous features, suggesting their origin in a jawed vertebrate ancestor.

## Aves: you only evolve germinal centers twice

Birds (Aves), the other vertebrate lineage to have evolved true endothermy, also exhibit specialized GC-like structures for B cell selection in their SLOs. Modern birds diverged from a common ancestor with other jawed vertebrates (specifically Reptilia) ~200 mya (54). While their SLO architecture shares similarities with mammalian white pulp, there are key anatomical differences in the organization of their lymphocyte zones.

The majority of our (limited) understanding of avian B cell selection is derived from studying the chicken (*Gallus gallus domesticus*) spleen, although there are reports that lymph nodes may be present in ducks (*Anas platyrhynchos domesticus*) (38). The chicken spleen contains defined lymphocyte regions, but they are not demarcated by a marginal zone as in the mouse spleen (**Figure 1**). A PALS surrounding the central arterioles contains T cells as well as reticular cells and macrophages (55). Adjacent to the PALS is a peri-ellipsoid sheath (PELS) at the termination of the splenic arterioles. This PELS region primarily contains B cells (55), and it is equivalent to the B cell follicles of the mammalian spleen. Between the PALS and PELS are additional white pulp regions containing observable lymphocytes, presumed to be a mixture of B and T cells (55).

**Figure 2:**
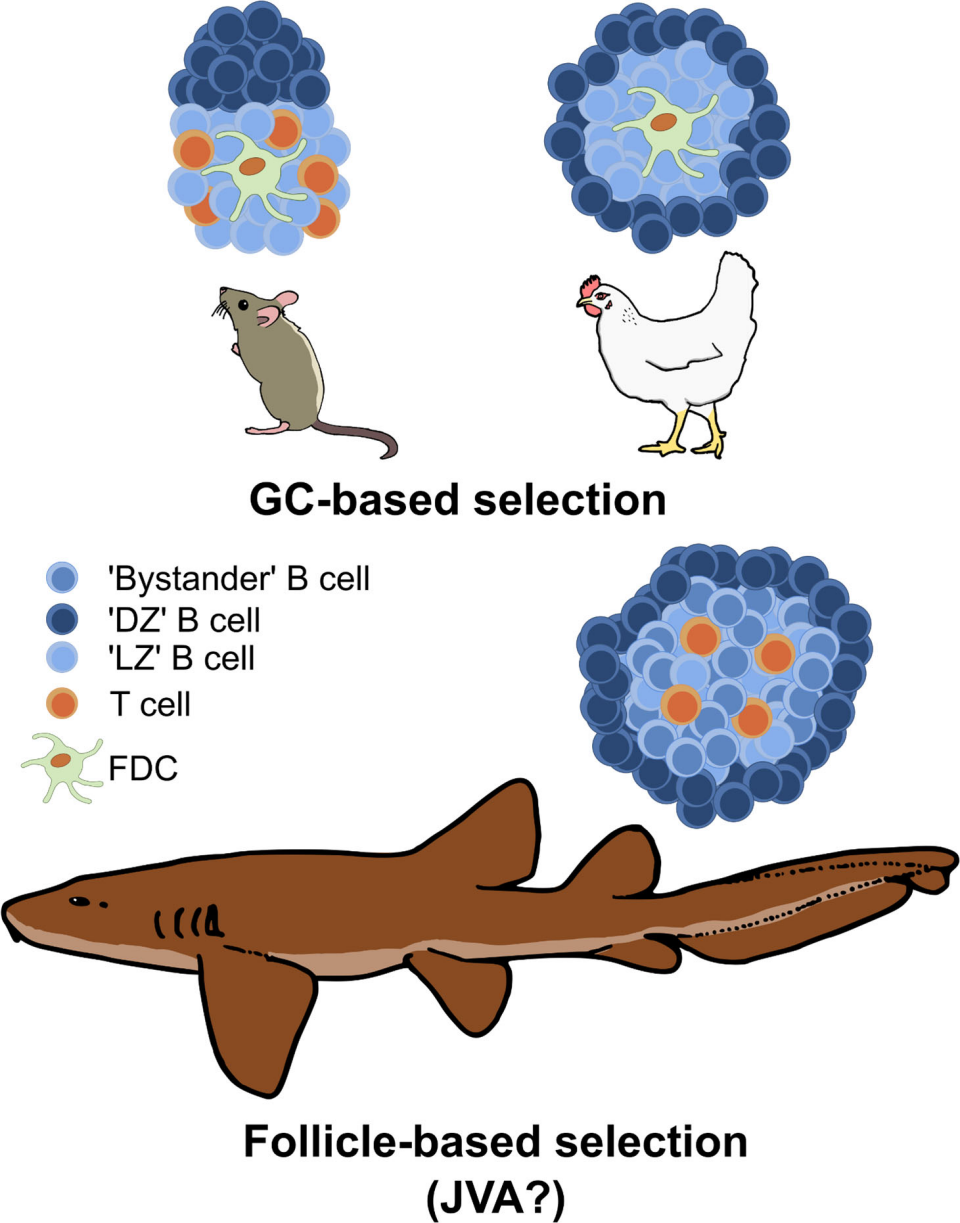
Comparison of GC structures in mammals and birds versus follicle-based selection in sharks. GCs segregate antigen-specific clones from the wider naïve B cell pool in the follicle, thus DZ and LZ B cells are predominantly derived from antigen-binding clonotypes. In contrast, B cell selection occurs across the entire follicle in sharks, with the central ‘LZ’ equivalent containing both ‘LZ’-like B cells (presumably antigen-specific) undergoing selection and ‘bystander’ B cells (non-antigen binding). This is hypothesized to produce a more ‘permissive’ but less efficient selection environment than found in GCs, and it may represent the primordial selection environment of the common jawed vertebrate ancestor (JVA). Cell types are annotated in the key.

Immunization with antigen produces GCs in the chicken spleen (55–57); these begin to form 3-4 days after immunization and are fully formed and discernable approximately 6-7 days post-immunization (55–57). While mammalian GCs form within the B cell follicle, chicken GCs form in the white pulp region between the PALS and PELS (55). In contrast to the mammalian GC which is divided into two poles, establishing the DZ and the LZ, Yasuda et al. showed the circumference of the chicken GC is the equivalent of the mammalian DZ (**Figure 2**). BrdU+ labeling in immunized chickens reveals that highly proliferative cells are localized around the outside of the GC in a ring-like formation. These cells are negative for surface Ig light (L) chain or IgM, similar to mammalian centroblasts in the DZ (55). The central region of the chicken GC is believed to be equivalent to the mammalian GC LZ, containing Ig+ positive cells as well as DCs able to trap ICs (55).

CD3+ T cells are also observed within the chicken GC, primarily within the central LZ (55). Observations suggest that the proportion of CD3+ T cells does not change within the chicken GC over the course of an immune response, but there is evidence for CSR and expansion of IgY+ (the avian ortholog of mammalian IgG) B cells (56). Immunization with labeled human serum albumin demonstrates that protein antigen begins to localize to the chicken white pulp within 2-3 days, but only becomes present within GCs 4-7 days after immunization (57). White et al. hypothesized that antigen localization in GCs was dependent on the formation of antibody-antigen ICs, as the increase in GC-localized antigen correlated with an increase in circulating antigen-specific antibody titers (57). In this study, antigen was retained in GCs up to 42 days post-immunization. The antigen-bearing cells within chicken GCs are believed to be true FDCs because hematopoietic lineage negative (CD45-) cells expressing surface VCAM-1, ICAM-1, and Ig (presumably as ICs) have been characterized (58). However, it remains to be confirmed if chicken FDCs are truly of a non-hematopoietic origin equivalent to mammalian FDCs.

The appearance of GCs in both mammals and birds is most likely a result of convergent evolution. No ectothermic vertebrates possess GCs, and birds share a more recent common ancestor with reptiles than with mammals, diverging approximately 300 mya (54). The absence of evidence for either GCs or endothermic metabolism before this divergence suggests these adaptations evolved at least twice in jawed vertebrates.

While different in cellular organization, avian GCs are functionally equivalent to mammalian GCs in their ability to select antigen-specific B cell clones. Increases in antigen-specific antibody titers are observed after immunization in birds (57, 59). Comparisons of antibody responses in rabbits versus chickens/ducks demonstrates that the avian B cell selection system can also increase the affinity of the polyclonal B cell repertoire (60, 61). In some animals, ongoing affinity maturation of the repertoire is observed after 50+ days, but this may be dependent on antigen availability (60). The avian Ig H isotypes are IgM and IgY, which comprise the majority of the primary and secondary responses, respectively, as well as IgA (reviewed in 62).

The endothermic jawed vertebrates thus define a paradigm of B cell adaptive immunity in which GCs are established early (2-4 days) after immunization, antigen-specific antibody titers increase relatively quickly, and affinity maturation results in significant (>100-fold) increases in antigen binding affinity.

## Reptiles: PALS and PELS are not enough (for robust affinity maturation)

Traveling further down the vertebrate evolutionary tree brings us to the non-avian reptiles (turtles, crocodilians, squamates, and tuatara), which diverged from other jawed vertebrates ~300 mya (54). Reptiles are the lineage with which birds most recently shared a common ancestor, so it is not surprising that the reptilian splenic structure closely resembles that of birds. Reptilian white pulp contains defined PALS and PELS regions (**Figure 1**). As examined in the Caspian turtle (*Mauremys caspica*), the inner region of the PELS appears to consist of Ig+ cells surrounded by an outer region of Ig- cells (63), presumably B cells and T cells respectively. The PALS appears to consist primarily of T cells (63). Variation in this microarchitecture is observed across various reptile species, with squamates (lizards and snakes) described as having less complex white pulp compared to chelonians (turtles) (63–65). Antigen-trapping DC-like cells have been observed in the white pulp of the reticulated python (*Malayopython reticulatus*) (64). Reptilian splenic white pulp also displays a ‘seasonality’ correlated to seasonal changes in thymic structure. The thymus, the primary lymphoid organ for T cell development in jawed vertebrates, undergoes involution seasonally in some reptiles, resulting in variations in the splenic microarchitecture (66, 67). Thymectomy (68) and adult thymic involution (69) also result in reduced cellularity of the PALS region of the reptilian spleen, further evidence that this region is an organized T cell zone. While reptiles have organized B and T cell zones, they do not form observable GCs in their white pulp (63, 64).

Despite this, reptiles are capable of generating antigen-specific Ig responses upon pathogenic infection or immunization (70–73). Evidence also exists for isotype switching and SHM in reptilian Igs (24). However, these antibody responses are slower than those observed in mammals and birds, with antibodies detected around one week post-immunization but titers typically not peaking until 6-8 weeks (22, 74, 75). True anamnestic immune responses have not been widely documented in reptiles. Reptilian antibody titers typically neither increase in magnitude nor attain peak levels more rapidly after secondary exposure to antigen (70, 72, 74, 76). Maturation of the binding affinity of the reptilian antibody response has also not been reported (70, 74). However, circulating antigen-specific antibody titers can persist in reptiles for significant periods after immunization (70, 71, 77). Origgi et al. investigated secondary challenge of Greek tortoises (*Testudo gracea*) with tortoise herpesviruses, reporting serum neutralizing antibody titers that may be indicative of a shorter latency period after re-exposure to the pathogen (22, 72). However, due to the absence of data indicating whether these antibody titers had returned to baseline prior to secondary challenge, it is not possible to definitively determine if these results prove true humoral immunological memory in this species. The Ig H chain isotypes expressed by reptilian B cells are IgM, IgD, IgY, the functional equivalent of mammalian IgG, and IgA depending on the species (reviewed in 78). Additionally, some reptile species have a unique isotype, IgD2, which is a chimera of IgD- and IgA-related constant domains and may play a role in mucosal protection (79, 80).

There is a paucity of literature on reptilian immune responses, making it difficult to draw definitive conclusions about this lineage. However, from the available data it seems that reptilian antibody responses lack the robust affinity maturation observed in endotherms as well as the rapid increases in secondary antibody titers. The slower humoral response in reptiles has been attributed to the lower core body temperature of ectotherms, but increasing the ambient temperature to 37°C did not alter the kinetics of the antibody response in turtles (70). It thus appears that while reptiles possess distinct splenic microarchitecture, the absence of GCs deprives their immune system of a selective environment capable of significantly maturing their antibody repertoires over the time periods studied.

## Amphibians: on double-duty antigen presenting service

Modern amphibians last shared a common ancestor with the other vertebrate groups ~350 mya (54) and represent the divergence point where tetrapods emerged and began to evolve terrestrial lifestyles. The best studied model of amphibian immunity is the African clawed frog (*Xenopus laevis*). In mammals, B cell follicles form around the central arterioles of the spleen during ontogeny and are later dispersed by the accumulation of T cells that form the PALS regions, establishing the mature splenic architecture. In *Xenopus*, B cell follicles also surround central arterioles, but there is no displacement to form observable T cell zones during development (**Figure 1**). Instead, the follicles are surrounded by a double layer of elongated cells termed the ‘Grenzschichtmembran of Sterba’ (GS) (81). T cells are dispersed outside this GS, but after immunization are recruited to the borders of the white pulp, presumably from the red pulp and peripheral circulation (82). The T cells form a corona surrounding the B cell follicle, and B cell-T cell contacts can be observed at the white pulp border (82). Thymectomy depletes lymphocytes from the red pulp and results in abolition of this T cell ring (83). AICDA is expressed in the follicles of the spleen after immunization (84), indicative of SHM. However, no GC-like structures or clusters of proliferating (BrdU+) cells have been observed in the spleen of *Xenopus* (85).

Amphibians are the first vertebrate class to have evolved canonical Ig CSR. The *Xenopus laevis* genome contains canonical switch (S)-regions to facilitate AICDA-mediated recombination (86). These S-regions can functionally replace mouse S-regions for CSR (87), and *Xenopus* AICDA can facilitate CSR in AICDA-deficient mammalian cells (88). It is possible that this adaptation co-evolved with the closer association of B cell-T cell interaction observed at the borders of the amphibian white pulp to facilitate efficient isotype switching. The amphibian Ig H chain isotypes as identified in *Xenopus* are IgM, IgD, IgX which plays an important role in mucosal immunity, IgY, and IgF (reviewed in 78).

APCs have been identified in amphibians that appear to fulfill the functional roles of both conventional dendritic cells (cDCs), presenting peptide antigens to T cells, and FDCs, presenting non-degraded antigen in ICs to B cells. These cells, first identified in *Xenopus laevis* and thus termed ‘XL’ cells, have the morphological characteristics of cDCs, including long cytoplasmic processes (89), and appear to be capable of trapping antigen at their plasma membrane (90). Following immunization with human IgG, antigen was found to localize at the perimeter of the *Xenopus laevis* white pulp, in a similar position to the XL cells (91). This led to the hypothesis that XL cells may be responsible for antigen trafficking and presentation in amphibian white pulp. This hypothesis was subsequently confirmed by Neely and colleagues who showed that in Xenopus immunized with fluorescent R-phycoerythrin (PE), antigen could be observed at the perimeter of the white pulp, where T cells subsequently colocalized (82). XL cells express both MHC class II and the T cell chemoattractant CCL19, indicative of a role in antigen presentation to T cells, but they are also capable of native antigen retention and presentation, exhibit positive surface staining for amphibian Igs, and express the B cell chemoattractant CXCL13 (82). Collectively, these results suggest XL cells may fulfill a ‘double-duty’ antigen presenting role for both T cells and B cells, preceding the evolution of canonical FDCs in endotherms.

The amphibian B cell response has similar kinetics to the reptilian response. IgM predominates the primary response for the first week or so, with IgY titers increasing approximately two weeks post-immunization, consistent with antigen-driven CSR (82). In *Xenopus* infected twice with the ranavirus Frog Virus 3 (FV3), FV3-specifc IgY titers were detectable around one week after the second infection, peaked at ~3 weeks, and remained detectable up to 4 weeks post-infection (92). These anti-FV3 antibodies could neutralize virus, and adult frogs challenged 15 months after infection displayed an anti-FV3 IgY memory response (93). *Xenopus* previously exposed to *Batrachochytrium dendrobatidis*, a chytrid fungus, exhibited pathogen-binding IgM, IgY, and IgX in mucus secretions (94), indicative of a role for Igs in amphibian mucosal immunity. However, the *Xenopus* IgY antigen-specific response to the hapten dinitrophenyl (DNP) displayed limited heterogeneity and affinity maturation compared to mammalian responses, hypothesized to be a result of ineffective B cell selection in the absence of GCs (20).

The amphibian adaptive immune system demonstrates a crossroads in evolution, where closer association of B cells and T cells in the splenic white pulp appears to be correlated with the emergence of bona fide CSR. ‘Double-duty’ APCs, capable of presenting antigen to both B cells and T cells, may define ectothermic B cell selection prior to the evolution of canonical FDCs and GCs.

## Teleosts: live and let macrophage

The most diverse lineage of vertebrates on Earth is by far the bony fishes (Osteichthyes), dominated by the ray-finned fishes (Actinopterygii) containing the infraclass teleosts (Teleostei). Ray-finned fishes diverged ~420 mya, with teleosts separating ~300 mya (95). Much of our knowledge of bony fish immunity has come from studying this group. The teleost spleen diverges markedly from the microarchitecture observed in the previously described jawed vertebrate taxa. There are regions of red pulp and white pulp, but lymphocytes are distributed throughout both without identifiable B cell follicles or major regions of B cell/T cell organization (96, 97) (**Figure 1**). Instead, following immunization or infection, lymphocytes appear to aggregate around pigmented macrophages, producing foci in the spleen known as melanomacrophage centers (MMCs) (96, 98–100). MMC presence and size appears correlated with infection status in bony fishes, and thus MMCs have been utilized to asses health in commercially relevant fish species (101). Melanomacrophages are highly phagocytic and will uptake injected materials, such as microspheres or bacteria (102, 103). There is evidence that antigen can be trapped in ICs in teleost spleen, although it remains unclear if these ICs are primarily retained by the MMCs or splenic ellipsoids (96, 104, 105). However, AICDA expression has been detected in cells interspersed within MMCs, suggesting a role for MMCs in the adaptive response (106). Additionally, cells within these clusters express Ig H chains with others expressing T cell receptors and CD4 (106).

Research in zebrafish (*Danio rerio*), the classic teleost model organism, by Waly et al. indicates that MMCs may function as a selective environment for B cell responses. Waly et al. found that individual MMCs display low BCR clonal diversity with expansion of oligoclonal lineages, suggesting proliferation of selected B cell clonotypes (107). They also found higher replacement to silent mutation ratios (R/S) in the complementarity determining regions (CDRs) of zebrafish Ig VDJ repertoires compared to the framework regions (FWR), indicative of antigen-driven selection favoring mutations in the antigen-binding region of Ig V sequences (107). Finally, they suggest that MMCs in the teleost spleen may trap and retain antigen (107) similar to mammalian FDCs. However, the role of MMCs as the de facto APC for B cell antigen retention and presentation in fish remains debatable. It remains possible that MMC formation is a physiological adaptation only tangentially associated with teleost immune responses (108). Further, whether teleost melanomacrophages produce B cell-recruiting cytokines such as CXCL13, or if they can also present antigen to T cells like *Xenopus* XL cells, remains to be determined.

There is an extensive body of literature examining teleost antigen-specific antibody responses, particularly in the context of vaccination against pathogens which threaten commercial aquaculture species (reviewed in 109). The teleost Ig H chain isotypes are IgM, IgD, and IgT (reviewed in 110). IgM responses dominate the teleost serum, while IgT is functionally equivalent to mammalian IgA, playing an important role in mucosal defense (111). Antigen-specific antibody titers can be detected in teleosts anywhere from 2-4 weeks post-immunization, with titers peaking at approximately 10-12 weeks post-immunization (18, 112). Depending on the immunizing antigen used, secondary responses can be detected in teleosts indicative of immunological memory (28, 113, 114). Affinity maturation of peripheral antibody responses has been detected in bony fishes. Cain et al. reported a 2-3 fold increase in antibody-antigen binding in immunized trout (18). Kaattari et al. demonstrated that this affinity maturation results from the emerging dominance of high affinity subpopulations of antibody later in the response (19), although it is not certain if this is a result of SHM of initially selected clonotypes or the expansion of newly selected clonotypes later in the response. The high affinity subpopulations persist longer and achieve higher titers than low affinity subpopulations (115). This shift to high affinity subpopulations was also observed in channel catfish *(Ictalurus punctaus)*, resulting in affinity maturation of the antigen-specific antibody response (112).

Considering the vast diversity of teleost species care must be taken not to generalize for the whole lineage using data obtained from only a few species. Indeed, the radiation of teleosts has resulted in extreme losses of adaptive immune function in several species. Genomic studies in Gadiformes (cod, haddock, pollock etc.) demonstrate a loss of both MHC class II and CD4 genes (116, 117), nullifying canonical T cell dependent antibody responses in this group. Additionally, cod AICDA is catalytically inactive, severely limiting secondary antibody diversification (118). The evolution of unique reproductive strategies also appears to have influenced the loss of immune genes in other bony fishes. Several genes of the MHC II pathway are lost in pipefishes (syngnathids) which exhibit male pregnancy (119), and some species of anglerfish (Lophiiformes), a lineage that has evolved extreme forms of sexual parasitism, have concomitantly lost many aspects of canonical adaptive immunity (120). These findings in teleosts underscore the malleability of adaptive immunity when confronted with singular evolutionary pressures.

While study of additional (varied) bony fish species will certainly improve our knowledge, current evidence supporting MMCs as the functional analogues of mammalian GCs in the bony fishes is far from conclusive. However, based on data from the other vertebrate lineages and recent studies performed in the more evolutionary ancient cartilaginous fishes (below), it is very unlikely that MMC-based selection represents the ‘primordial’ state found in the jawed vertebrate ancestor, but is more likely a bony fish-specific derivation.

## Cartilaginous fishes: for B cell follicles only

Cartilaginous fishes (Chondrichthyes) are the oldest extant taxonomic group of vertebrates with Ig-based adaptive immunity. Chondrichthyes emerged in the early Silurian period ~450 mya (121, 122) and is divided into two subclasses: Holocephali (chimeras or ratfishes) and Elasmobranchii (sharks, skate, and rays). The elasmobranchs have been the focus of most studies on cartilaginous fish immunity; in particular, the little skate (*Leucoraja erinacea*) and nurse shark (*Ginglymostoma cirratum*) have been key model organisms for this lineage.

The tissue architecture of the nurse shark spleen reflects the basal position of this lineage in phylogeny. There are identifiable red pulp and white pulp regions, but no marginal zone or border surrounding the white pulp (123) (**Figure 1**). During development, B cells begin to populate the spleen around the central arterioles, forming nascent follicles (124). In adult animals, Ig+ B cells populate the follicles of the white pulp, and Ig secreting plasma cells are identifiable in the red pulp (125). Cells with dendritic-like processes have also been observed in nurse shark spleen which express MHC class II and may accumulate antigen on their plasma membranes (39, 124). No identifiable T cell zones are present in the shark spleen, and up until recently it was unclear how T cells were organized in cartilaginous fish SLOs. *In situ* hybridization experiments revealed T cells are found in small aggregates within the red pulp, external to the B cell follicles (126). Visually discernable GCs have not been found in the shark spleen (16, 123).

Our laboratory recently provided the first evidence for how B cell selection may be orchestrated within the cartilaginous fish SLO. Fluorescence *in situ* hybridization experiments revealed that the splenic follicles of immunized nurse sharks contain Ig+/AICDA+/Ki-67+ cells around the circumference of the white pulp (127) (**Figure 2**). CD3ϵ+ T cells were identified in aggregates associated with the borders of B cell follicles, as well as distributed within the follicles themselves (127). Single nuclei sequencing of nurse shark spleen samples revealed B cells with centrocyte- and centroblast-like gene expression signatures (127). Additionally, we identified a subset of T cells that expressed genes associated with mammalian Tfh cells (127). Finally, we demonstrated that nondegraded antigen in the form of fluorescent R-PE can be presented in the center of B cell follicles, and the Ig V regions of BCR sequences isolated from follicles show higher R/S ratios in the CDRs compared to FWRs (127). Collectively, these results suggest shark B cells undergo selection against antigen in the center of follicles, possibly receiving co-stimulation from Tfh-like cells. They then migrate to the outer edge of the follicle to proliferate/hypermutate, and eventually exit the follicle to differentiate into antibody-secreting plasma cells. Of note, the organization of the immunized shark B cell follicle with proliferating cells around the circumference resembles the structure of the avian GCs identified by BrdU+ staining (55) (**Figure 2**).

The presence of nondegraded antigen retained in shark B cell follicles suggests that the paradigm of ‘double-duty’ presenting cells may have evolved earlier than XL cells in amphibians. FDCs have not been identified in any ectothermic vertebrates, but more definitive work is necessary to determine if cartilaginous fishes have APC populations capable of presenting antigen to both T cells in an MHC class II dependent fashion, as well as B cells in the form of captured ICs. Certainly, the accumulating data demonstrates that the antigen presenting function of FDCs was present in jawed vertebrates long before the cell type emerged in mammals (82, 127).

Despite the lack of complexity in cartilaginous splenic architecture compared to mammals, the ‘B cell follicle only’ system still supports robust humoral immunity. Sharks have 3 Ig H chain isotypes: IgM, expressed in pentameric (pIgM) and monomeric (mIgM) forms; IgW, the ortholog of mammalian IgD; and Ig new antigen receptor (IgNAR), an H-chain only isotype that does not associate with L chain (reviewed in 128). Early antibody responses are dominated by low affinity, high avidity pIgM (21). After immunization, antigen-specific mIgM and IgNAR titers increase for up to 2-3 months and persist for long periods (21). Shark Ig genes mutate at very high rates (26, 129), and both mIgM and IgNAR undergo affinity maturation (17, 21). Humoral immunological memory has been described in nurse shark up to 8 years after primary exposure (31).

Even in cartilaginous fishes, a lineage that diverged very early in jawed vertebrate evolution, key hallmarks of GC-based B cell selection are present in the SLO.

## No time for slow selection—why did endotherms evolve germinal centers?

From the studies highlighted above, two conclusions emerge. First, SLO architecture increases in complexity as one moves forward in jawed vertebrate evolution, culminating in the emergence of GCs in birds and mammals (**Figure 1**). The exception to this paradigm is the bony fishes, which lack discernable white pulp or organized lymphocyte zones. Second, the major functional characteristics of humoral immunity, specifically the capacity to produce antigen-specific antibody titers, to affinity mature antibody repertoires, and to generate secondary memory responses, are present in all jawed vertebrate lineages to some extent. If these key immunological adaptations are possible without GCs, it begs us to ask why did the specialized microstructure of the GC evolve? Based upon current data it seems that the emergence of GCs is not due to the presence of a more ‘advanced’ adaptive immune system in endothermic jawed vertebrates. Rather, the convergent evolution of endothermic metabolism in birds and mammals may have necessitated the development of a more stringent B cell selection environment in these lineages.

Cells able to present antigen to B cells are clearly present in ectothermic vertebrates even if bona fide FDCs are not. Interestingly, several lines of evidence demonstrate that some mammalian cDCs can present membrane-bound antigen to B cells (reviewed in 130). Specifically, the type 2 cDC subset (cDC2) which localizes to the B-T cell border may exhibit ‘double-duty’ abilities and present antigen to both B cells and T cells in mammals (131, 132). Indeed, such cDCs could be the primordial system of antigen display, with FDCs co-opting this role very late in jawed vertebrate evolution.

Our recent work shows that intact antigen is widely distributed across the center (and apparent LZ equivalent) of nurse shark B cell follicles (127). Given that B cell division rates are almost certainly slower at lower (i.e., exothermic) body temperatures and there is no visible segregation of antigen-specific clones from the wider population of B cells, we hypothesize that this reduces selection pressure, facilitating the retention of lower affinity clones while also supporting the production of an extensive and diverse memory B cell pool (133). Such a permissive selection environment would explain the delayed antibody responses and lower levels of affinity maturation reported in cartilaginous fishes and other ectothermic vertebrates.

In contrast, GCs in endotherms are founded by antigen-specific B cell clones that have received T cell help. These B cells can divide at a much faster rate at an endothermic core body temperature, and so the selective environment becomes increasingly segregated from the wider pool of non-specific clones. Further, only the FDCs at the center of the LZ act as a long-term reservoir of intact antigen in mice (134). Thus, it appears that the distinct microanatomical structure of the GC intensifies selection pressures by increasing competition for antigen and T cell help, thereby facilitating faster, more efficient selection of higher affinity antigen-specific B cells in endotherms. This would be evolutionary advantageous given that the maintenance of warmer core temperatures also supports the rapid proliferation of potentially infectious organisms (135–137), and may be required in endothermic vertebrates to avoid succumbing to pathogen infections before sufficient protective antibody titers can be mounted (138).

However, ‘faster’ selection does not, on its own, appear to fully explain the evolutionary advantage of GCs (138). While endotherms attain peak antibody titers more quickly than ectotherms (138), pathogens generally exhibit far shorter replicative cycles than the host organisms they infect, whether endothermic or exothermic. Influenza-like viruses, for example, infect a wide range of endothermic and ectothermic vertebrate hosts and successfully replicate at a variety of temperatures (139–141). While viruses in ectotherms may replicate slower than in endotherms, surely those pathogens which escape immediate control by the innate immune system still pose a threat to host survival long before antibody responses begin to peak. Under this lens, antibody-based adaptive immunity would bestow little evolutionary advantage.

Rather, we propose that the initial selective advantage of antibody-based adaptive responses was the immunological memory conferred by antigen-experienced B cell clones. In this scenario an ectothermic jawed vertebrate ancestor with a ‘slow-but-permissive’ adaptive immune system could produce a diverse memory repertoire capable of combating future pathogen variants. Indeed, it has been suggested that the primordial role of SHM was to further diversify the repertoire of antigen-specific memory B cells (142). When endothermic metabolism emerged as an adaptation it accelerated B cell division in SLO selective sites, resulting in visually discernable GCs able to perform B cell selection quicker and more stringently. While not the driving force, a by-product of this evolutionary process would be more rapid primary responses and higher levels of affinity maturation, as observed in endotherms. Indeed, a possible drawback of faster B cell selection is immunodominance (reviewed in 143), leading to vaccine responses predominantly directed towards undesirable epitopes. Restrictive selection may also result in a low frequency of *de novo* clones responding to heterologous antigens (144, 145), thus limiting the final repertoire.

Understanding that the mammalian GC response is just an accelerated version of the selection models in ectotherms suggests that vaccine efficacy may be improved by modifying immunization strategies. Indeed, strategies such as slow release of antigen (146) or promotion of persistent GCs (147), mimicking the selection environment/kinetics in ectothermic vertebrates, seem to generate more diverse B cell responses better able to deal with pathogen variants.

## Conclusion

It is increasingly apparent that sophisticated B cell selection mechanisms incorporating T cell help, SHM driven by AICDA, and specialized cells capable of presenting native antigen emerged early in jawed vertebrate evolution. As evolutionary pressures forced more efficient selection of antigen-specific B cell clones, the complexity of SLO tissue organization also increased to facilitate the coordination of B cell, T cell, and APC interactions, culminating in the appearance of GCs. While mammalian GC selection may be more stringent, this may come at the cost of antigenic imprinting and B cell immunodominance. New immunization strategies that mimic the selective environment/kinetics of ectothermic vertebrates may help in the fight against rapidly evolving pathogens such as influenza, HIV, or human coronaviruses.

## Author contributions

HM and HD conceived the general topic of the review. HM wrote the manuscript and designed the figures. HD provided insightful feedback and criticism of the final manuscript.
